# A Low-Cost Protocol Using the Adjunctive Action of Povidone–Iodine Irrigations and Sodium Hypochlorite Rinsing Solution in Step 2 of Periodontal Therapy for Patients with Stage III–IV Periodontitis: A Single-Blind, Randomized Controlled Trial

**DOI:** 10.3390/dj12050144

**Published:** 2024-05-15

**Authors:** Georgios Kardaras, Ruxandra Christodorescu, Marius Boariu, Darian Rusu, Alla Belova, Salvatore Chinnici, Octavia Vela, Viorelia Radulescu, Simina Boia, Stefan-Ioan Stratul

**Affiliations:** 1 Department of Periodontology, Faculty of Dental Medicine, Anton Sculean Research Center for Periodontal and Peri-Implant Diseases, “Victor Babes” University of Medicine and Pharmacy, 300041 Timisoara, Romania; kardaras.georgios@umft.ro (G.K.); rusu.darian@umft.ro (D.R.); alla.belova@umft.ro (A.B.); salvatore.chinnici@umft.ro (S.C.); vela.octavia@umft.ro (O.V.); viorelia.radulescu@umft.ro (V.R.); simina.boia@umft.ro (S.B.); stratul.stefan@umft.ro (S.-I.S.); 2 Department V Internal Medicine, Faculty of Medicine, “Victor Babes” University of Medicine and Pharmacy, 300041 Timisoara, Romania; christodorescu.ruxandra@umft.ro; 3 Department of Endodontics, Faculty of Dental Medicine, TADERP Research Center, “Victor Babes” University of Medicine and Pharmacy, 300041 Timisoara, Romania

**Keywords:** periodontitis, antiseptics, antivirals, antibiotics

## Abstract

In severe stages of periodontitis, conventional periodontal therapy and maintenance care are usually insufficient due to the viral and bacterial etiology; thus, a mechanical approach alone may not be sufficient to eliminate a substantial portion of subgingival pathogens, especially in deep periodontal sites. *Background and Objectives*: This single-blind, randomized clinical trial aimed to compare the clinical and microbiological efficacy of a low-cost protocol using povidone–iodine and sodium hypochlorite formulations as adjuncts to non-surgical therapy for patients with stage IV periodontitis when compared with chlorhexidine, the most commonly employed substance to date for antimicrobial regimens in periodontal therapy. *Materials and Methods*: Forty-five patients were randomly divided into two groups: control (subgingival instrumentation, chlorhexidine-assisted) and test (antiviral medication, subgingival instrumentation with povidone–iodine, sodium hypochlorite rinsing solution, and antibiotics). Clinical measurements and microbiological analyses were performed at baseline and after three months. *Results*: After three months, notable differences were found in the bacterial detection scores for *Porphyromonas gingivalis* (a significant reduction in detection frequency was observed in the test compared to the control (*p* = 0.021)), and there were significant reductions in detection in the test group for *Tannerella forsythia* and *Treponema denticola*, showing undetectable levels (*p* < 0.0001 for both). In the test group, the pocket probing depth median value was reduced significantly (*p* = 0.0005); similarly, bleeding on probing showed a marked decrease (*p* < 0.0001). However, changes in clinical attachment loss and full-mouth plaque score were not statistically significant. *Conclusions*: Using the proposed protocol, substantial improvements in clinical and microbiological parameters were obtained when compared with the current antimicrobial recommendations.

## 1. Introduction

Periodontal disease is a long-term, multifactorial inflammatory condition associated with dysbiotic plaque biofilms, bacterial infections, and host immune responses that create a distinct microenvironment that affects periodontal homeostasis [1]. Generalized severe periodontitis (stage III and IV, with an interdental CAL of ≥5 mm at the site of greatest loss) affects multiple teeth, which implies a high risk of edentulism and tends to occur in individuals who exhibit high loads of periodontal pathogens; it is associated with major risk factors or serious systemic diseases [2,3].

Several authors assume that virtually all cases of aggressive periodontitis involve an active herpesvirus infection, which has the potential to impair periodontal host defenses and induce an overgrowth of pathogenic bacteria [4,5,6,7,8,9,10,11,12]. Viral DNA has been detected in gingival tissue, gingival crevicular fluid, and subgingival plaque from periodontally affected sites [13]. Periodontal destruction can be influenced by the cohabitation of viruses like periodontal human cytomegalovirus or Epstein–Barr virus and perio-pathogenic bacteria and precarious local host immune responses [14]. Active human cytomegalovirus replication in periodontal sites suggests that its re-activation triggers periodontal disease activity [13].

As recommended by the recent European Federation of Periodontology S3 level Clinical Practice Guideline (CPG) for the Treatment of Stage I–III Periodontitis, the second step of therapy is aimed at controlling the subgingival biofilm and calculus and includes subgingival instrumentation and the use of adjunctive physical and chemical agents, with chlorhexidine digluconate being the gold-standard antiseptic [15,16]. As recommended by the CPG, the adjunctive use of specific systemic antibiotics may be considered for specific patient categories, like stage III periodontitis, considering the global concerns regarding the overuse of antibiotics and the development of antibiotic resistance [15].

As periodontitis is a major health problem in low-income populations, the successful periodontal healthcare of such populations must be based on affordable professional therapy and self-care techniques with proven efficacy and safety [17,18,19,20]. The self-management of periodontal disease is of the utmost importance for individuals with limited access to professional hygiene [21]. As relating the change in financial costs to the change in therapeutic outcome [22] is the basic approach to allocating healthcare resources most efficiently, periodontitis therapy should consider reducing costs by using established, low-cost, yet highly effective antimicrobials.

A low-cost antimicrobial, 7.5–10% povidone–iodine (PVP–I), used as subgingival irrigation during step 2, has been used to treat periodontitis [23,24,25,26,27] and in re-treatment during step 4 [23]. The literature recommends subgingival irrigation with 10% povidone–iodine for 5 min to reduce bacteremia before mechanical debridement to eradicate any remaining pathogens [27,28]. It exhibits antiseptic action by eradicating most oral microorganisms (perio-pathogens, fungi, mycobacteria, viruses, and protozoa) with no cytotoxic effects on human cells and by destabilizing bacterial lipid membranes and proteins [29]. Relatively similar protocols based on the use of another low-cost antimicrobial, sodium hypochlorite, as a periodontal disinfectant were proposed. The delivery method varies according to the step of periodontal therapy [15] and the form of use. NaOCl was used as a 0.05–0.25% rinsing solution in step 1 [23,24,25] and as subgingival irrigation in step 2, with concentrations varying from 0.5% [26,27] to very high concentrations of 5.6% [28,30]. In addition, in recent years, NaOCl as a 0.95% gel has been used in the treatment of periodontal [31,32] and peri-implant infections [33] during steps 2 and 4 [34].

Since 2002, both antimicrobials have been proposed in protocols that provide affordable, active, and competent antimicrobial therapy, intended to result in high antimicrobial efficacy, while maintaining low costs of periodontal therapy [17,35,36,37,38]. This protocol has been modified over the years by its authors regarding the NaOCl concentrations (0.05%, 0.10%, 0.2%, 0.25%), as the modifications are guided by the individual acceptability of the taste, smell, and strength of the solutions by the patients [17,36,37,38]. Currently, there are no available patient-centered clear data regarding the aforementioned individual perception.

The latest version of this protocol, stretching over steps 1, 2, and 4 of periodontal therapy, includes anti-infective substances that target herpesviruses (valacyclovir) and perio-pathogenic bacteria (amoxicillin + metronidazole) and enrolls the common, low-cost, effective antiseptics, povidone–iodine and sodium hypochlorite, in addition to scaling [38,39]. Its rationale is that active herpesviruses and specific pathogenic bacteria are involved in the etiology of severe periodontitis [36,37,38,39], and a potent and affordable anti-infective treatment targeted to reduce or eradicate all these pathogens is needed [36,40].

To our knowledge, elements of this protocol have been used in numerous studies [2,4,17,39,40,41,42] with various outcomes; however, the comprehensive concept of the proposed periodontal therapy was not the subject of any longitudinal controlled clinical trial.

The present study aimed to evaluate the clinical and microbiological changes following a therapeutic protocol including povidone–iodine irrigations and the administration of antiviral medication during step 1, sodium hypochlorite home-care rinsing, and systemic antibiotics after step 2 of therapy in patients with severe periodontitis, when compared with the same treatment sequence, but without antiviral intake and using chlorhexidine solution as an adjunctive antimicrobial. Patient-centered data regarding the patient’s perception of the sodium hypochlorite solution used for rinsing were also collected and analyzed. The null hypothesis of this study is that a low-cost regimen of potent local antimicrobials combined with systemic antibiotics adjunctively administered to subgingival instrumentation in the treatment of severe periodontitis does not result in improved clinical and microbiological parameters when compared to the chlorhexidine gold standard.

## 2. Materials and Methods

### 2.1. Study Design

**Figure 1:**
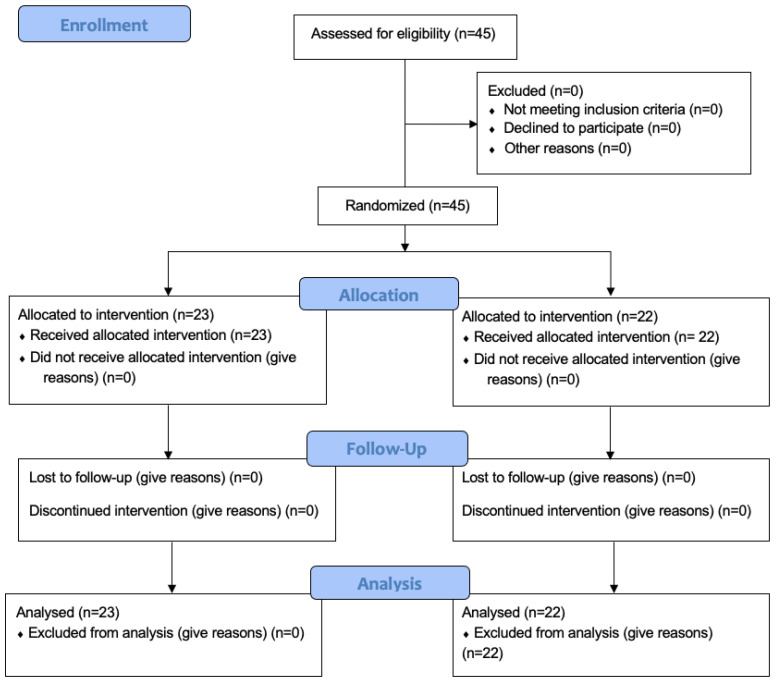
CONSORT flow chart.

Between February 2022 and June 2023, this single-blind, randomized, controlled clinical trial of 3 months with a parallel design of two independent groups with a 1:1 allocation ratio enrolled 45 subjects—outpatients at the Clinic of Periodontology of the Faculty of Dental Medicine of the “Victor Babeş” University of Medicine and Pharmacy Timișoara, Romania. The single-blind design was chosen due to the physical properties (color, taste, and smell) of the antimicrobial products, which make them easily distinguishable. The study protocol was approved by the Research Ethics Committee of the “Victor Babeş” University of Medicine and Pharmacy (approval No. 56/2020). The study was registered in the ISRCTN10470122 Registry of Clinical Trials and followed the guidelines described in the CONSORT 2010 (Consolidated Standards of Reporting Trials) statement on clinical trials (Figure 1). The blinding was achieved by blinding the examiners.

The study was conducted over 15 months (February 2022–November 2023), and the procedures followed were in accordance with the Declaration of Helsinki, the ethical standards of the responsible committee on human experimentation, and the rules of good practice in biomedical and educational research. All patients were informed about the nature and purpose of the study, and each of them signed an informed consent document granting permission for the dental procedures and sampling of biological material.

### 2.2. Study Population

The study population consisted of males and females (mean age, 48.3 ± 9.1 years; range, 33–71 years). The study eligibility criteria included systematically healthy patients with a clinical diagnosis of periodontitis stage III or IV and grade B or C, according to the Classification of Periodontal and Peri-implant Diseases and Conditions 2018 [43].

The criteria for exclusion were patients who had received antibiotic, anticoagulant, or immunosuppressive therapy during the preceding 6 months; patients who used oral antiseptics or received any periodontal therapy; pregnant or lactating women; patients in need of antibiotic prophylaxis during the preceding six months; patients with known allergies and hypersensitivity to PVP–iodine and NaOCl; those with thyroid dysfunction; and patients with allergies to the recommended medications or systemic disorders. Lastly, smokers were excluded from the current study to avoid the potential confounding effects of cigarette smoking.

### 2.3. Calibration

The study team included two examiners (experienced specialists in periodontology), a randomizer, and two operators (specialists in periodontology). To perform the inter-examiner calibration, five patients with severe periodontitis (not included in the study) were selected. The inter-examiner value of agreement in measuring the PPD and CAL in these patients was 93% and 88%, respectively, for a difference of <1 mm, for more than 97% of the examined periodontal sites.

### 2.4. Clinical Measurements

Emergency care was delivered where necessary. Prior to evaluation and treatment, retentive restorations were removed, and cavities were filled. Information about periodontal disease and instructions for performing proper oral hygiene were given to the patients. An electric toothbrush was recommended, and dental floss and interproximal brushes were used to demonstrate how to perform interdental care. The oral hygiene instructions were repeated both during the basic treatment period and at each recall visit. A panoramic dental X-ray was obtained at the first consultation. After this first visit, teeth that were considered hopeless were extracted.

The clinical baseline evaluation and the three-month re-evaluations were performed by the same two previously calibrated examiners who were unaware of the treatment allocation. After the assessment of the full-mouth plaque score (FMPS), as well as the full-mouth bleeding score (FMBS), the pocket depths (PDs) and recessions (RECs) were documented to analyze the PD reductions as the primary outcome variable, while CAL gain, REC, FMBS, and FMPS were regarded as secondary outcomes. Full-mouth plaque scores (FMPSs) were recorded using six sites per tooth. The presence of plaque was determined using a disclosure solution (Dentorama Blue Disclosing Pellets Pro-155, Svenska Dentorama AB, Solna/Stockholm, Sweden) in a dichotomic way, with regard to the percentage of sites with plaque [44]. Bleeding on probing (BOP) was assessed dichotomously in 6 sites per tooth, using a periodontal probe (Force-Control Periodontal Probe WHO DB765R, Aesculap, Tuttlingen, Germany) to calculate the full-mouth percentage of the bleeding score (FMBS). The method of calculation was the same as for FMPS. Pocket depths (PPDs) were measured on six sites per tooth. Recessions (RECs) were recorded to the nearest millimeter at six sites per tooth. The clinical attachment level (CAL) was calculated using the PD and REC values. Measurements were rounded up to the next whole-millimeter value. Mobility was recorded in degrees, according to the Miller classification system (1985) [41]. The aforementioned periodontal parameters were recorded in the periodontal chart (http://www.periodontalchart-online.com/uk/, URL accessed on 1 February 2022), printed, and included in each patient’s observation file.

### 2.5. Microbiological Sampling

In the same appointment, bacterial samples were taken as described below: to detect the 5 major keystone bacteria, Aggregatibacter actinomycetemcomitans (Aa), Porphyromonas gingivalis (Pg), Prevotella intermedia (Pi), Tannerella forsythia (Tf), and Treponema denticola (Td), a molecular genetic analysis was performed using the commercial kit micro-IDent plus (Hain Lifescience GmbH, Nehren, Germany). During the initial evaluation, samples of subgingival plaque were collected from the deepest periodontal pockets in each quadrant with four sterile paper points #25 (ProTaper Next^®^ Paper Points X2; Dentsply Sirona, Charlotte, NC, USA) inserted into the gingival sulcus and used to identify the existing bacterial strains prior to treatment at the three-month re-evaluation to assess post-treatment bacterial suppression.

An antimicrobial regimen including ultrasonic and manual scaling and adjunctive use of 10% betadine, acyclovir intake, and 0.2% hypochlorite rinsing as home care, according to the protocol of Slots 2020 [36], was implemented in the test group, while in the control group, the antimicrobial substance used was chlorhexidine 0.2%.

### 2.6. Patient-Centered Data Collection

Data regarding the perceptions of the recommended concentration of hypochlorite were collected using a VAS scale. Perception of the taste during use, tolerance, oral dryness sensation, and aesthetic impact of staining after three months of use were evaluated by the patient on a scale from 1 to 10. Other subjective perceptions regarding taste modifications, oral mucosa irritations, long-term negative effects, the intensity of the sensation of cleanliness, the ease of solution preparation, and economic impact were collected and evaluated on a 1 to 5 scale of approval.

### 2.7. Randomization

Patients were allocated into two groups using a list compiled in advance with the randomization software www.random.org, accessed on 1 February 2022. The computerized randomization assigned the patients to one of the groups, with an allocation ratio of 1:1. The randomizer performed the assignment to interventions, while a dental assistant performed the documentation. An allocation table was created and used to assign each patient a treatment number.

### 2.8. Periodontal Treatment

#### 2.8.1. Test Group

On day 0, 10% povidone–iodine (undiluted 10% Betadine^®^, Egis Pharmaceuticals, Budapest, Hungary yielding 1% free iodine) was used in subgingival irrigation for a total of 5 min. Irrigation was performed with a 10 mL sterile plastic syringe and blunt needle (0.6 mm diameter, Ultradent Products Inc., Cologne, Germany) placed about 1 mm coronally to the bottom of the pocket. Each course of subgingival povidone–iodine irrigation took a minimum of 90 s for the entire dentition and was performed 3 times for a total application time of 5 min [36]. Full-mouth gross scaling with ultrasonic instruments was performed to reduce subgingival calculus and to diminish gingival bleeding. Ultrasonic scalers were used (instrument A, EMS Piezon^®^ Master, EMS, Nyon, Switzerland). When necessary, local anesthesia was performed. To eliminate high loads of herpesviruses in deep periodontal pockets and within inflamed gingiva, acyclovir (500 mg, twice daily for 10 d) was prescribed in the same appointment and the administration lasted from day 0 to day 10. Patients were warned about the most common adverse events of acyclovir, like nausea, stomach pain, headache, and dizziness. Patients were instructed to perform self-care with freshly prepared sodium hypochlorite twice weekly, in 30 s oral rinses. Sodium hypochlorite was available as medical-grade formulations (Chloraxid 5.25%, Cerkamed, Stalowa Wola, Poland) and was preferred over household bleach because of the likelihood of a precise dosage at the desired concentration. The original package is an opaque container of either 200 or 400 mL, with a special cap allowing graded syringes to be safely screwed in. Patients were given 100 mL shaded plastic bottles to facilitate the preparation of the desired antimicrobial concentration. As 0.2% seems to be the concentration of choice for most patients, 0.2% sodium hypochlorite can be obtained for self-care by adding 1 mL of 5.25% Chloraxid to ab. 26 mL of tap water. Patients were instructed to rinse using one cap of the prepared solution. Patients were instructed to refrain from using all other antiseptic solutions during home care. On day 10, follow-up subgingival instrumentation was performed only in sites with verified calculus or suspicion of calculus, as antimicrobials are expected to remove perio-pathogens in sites that have not been scaled [45]. The periodontal treatment was performed by an experienced clinician (specialist in periodontology). Amoxicillin + metronidazole (250 mg of each, 3 times daily for 8 d) for young and middle-aged patients or ciprofloxacin + metronidazole (500 mg of each, twice daily for 8 d) for older patients and patients allergic to penicillin were prescribed. Patients were warned about common adverse events related to the intake of the prescribed antibiotics. To reduce dental biofilm buildup and to prevent gingival inflammation, patients were instructed to perform self-care in the same way as before, using freshly prepared 0.2% sodium hypochlorite twice weekly in 30 s oral rinses, for the long term.

#### 2.8.2. Control Group

In the control group, patients received the same treatment, except they did not receive antiviral and antibiotic medication, and 10% PVP–iodine solution was replaced by 0.12% chlorhexidine solution (Dentaton, Ghimas, Casalecchio di Reno, Italy), while self-care rinsing at home with 0.2% hypochlorite solution was replaced by rinsing with 0.12% chlorhexidine solution.

### 2.9. Statistical Analysis

In this single-blind, randomized, controlled clinical trial, the sample size was determined by a convenience sampling method noted in a similar previous study [46].

Changes between appointments (Δ) were calculated as the difference between the initial and final values of each parameter. The primary outcome was the intergroup difference in the reduction in PPD, while CAL, FMPS, FMBS, and the microbiological parameters were considered secondary outcomes. It was determined that 14 patients per group would be needed to achieve 80% power to detect a significant mean difference of 1 mm in the reduction in PPD between groups (assuming a common SD of 0.75 mm and given significance level alpha = 0.05).

Python v.3.8 software (Python Software Foundation, Wilmington, DE, USA) was used. Intragroup comparisons used paired *t*-tests for Gaussian data, and intergroup comparisons used unpaired *t*-tests. For non-Gaussian data, median values between groups were compared using the Mann–Whitney U test. The chi-square test was employed to compare the proportions between the two study groups. Correlation analyses using Pearson’s rho and logistic regression analyses were conducted to examine relationships between clinical measurements and bacterial detection. The study power was considered for 80%, with a significance level of 0.05. The Shapiro–Wilk test was used to test the normality of data.

Detection frequency scores were utilized to quantify the presence and intensity of specific bacterial pathogens at baseline and three months post-treatment. The scoring system ranged from 0 to 4, where “0” indicated undetectable levels; “1” mild detection—10^4^ (10^3^ for *Aa*); “2” moderate detection—10^4^–10^5^ (10^3^– 10^4^ for *Aa)*; and “3 and 4” high detection—3—10^5^– 10^6^ (10^4^–10^5^ for *Aa*), 4—>10^7^ (10^6^ for *Aa*). Statistical analysis was performed to compare detection scores within groups using the Wilcoxon test and between groups using the Kruskal–Wallis and Mann–Whitney tests.

Statistical analysis of the patient-centered data was conducted using IBM SPSS v.27 software. For the quantitative data, descriptive statistics were initially employed to summarize the background data: the calculation of medians, interquartile ranges (IQRs), means, and standard deviations (SDs). To assess the differences in perceptions based on age and gender, inferential statistics were applied. The Mann–Whitney U test was used to analyze age-based comparisons. For gender-based comparisons, independent sample *t*-tests were conducted.

## 3. Results

The patient demographics are presented in Table 1. No statistically significant differences between the groups were found regarding the mean age and the sex distribution.

At baseline, there were no statistically significant differences for the clinical parameters of PPD, CAL, FMPS, and BOP among groups. The distributions of bacterial detection scores (*Aa*, *Pi*, *Pg*, *Tf*, *Td*) among the groups also showed no statistically significant differences (Table 2).

The PPD and CAL measurements at 3 months did not differ significantly between the control and test groups, with PPD showing means of 3.53 and 3.60 mm (*p* = 0.7774) and CAL showing means of −4.50 and −4.61 mm (*p*-value = 0.7083), respectively. Similarly, the FMPS and BOP percentages also did not differ significantly, as well as the BOP means of 16.57% and 21.23% (*p* = 0.3434) for the control and test, respectively.

However, notable differences were found in the bacterial detection scores for *Pg*, *Tf*, and *Td*. In the case of *Pg*, a significant reduction in detection frequency was observed in the test compared to the control, with 86% of the test group undetectable for *Pg* at 3 months (*p* = 0.0210). For *Tf* and *Td*, there were significant reductions in detection in the test group, with 64% and 59% of participants, respectively, showing undetectable levels (*p* < 0.0001 for both). This contrasted with controls, where 39% and 57% of the participants still showed moderate to high detection for *Tf* and *Td*, respectively. In contrast, *Aa* and *Pi* did not exhibit statistically significant differences in detection scores between the groups (*p* = 0.2241 and 0.1663, respectively) (Table 2).

At 3 months, significant improvements were observed in the control group (n = 23) across all measured variables. The PPD median decreased from 4.5 to 3.3 mm (*p* < 0.0001), reflecting a notable reduction. CAL also showed a significant improvement, with the median moving from −5.3 to −4.5 mm (*p* < 0.0001), suggesting a gain in clinical attachment. The FMPS median value decreased from 15.0 to 7.0 (*p* = 0.0364), indicating improved plaque control. A significant reduction in BOP was also noted, with the median decreasing from 63.0 to 15.0 (*p* < 0.0001), indicative of reduced gingival inflammation.

**Figure 2:**
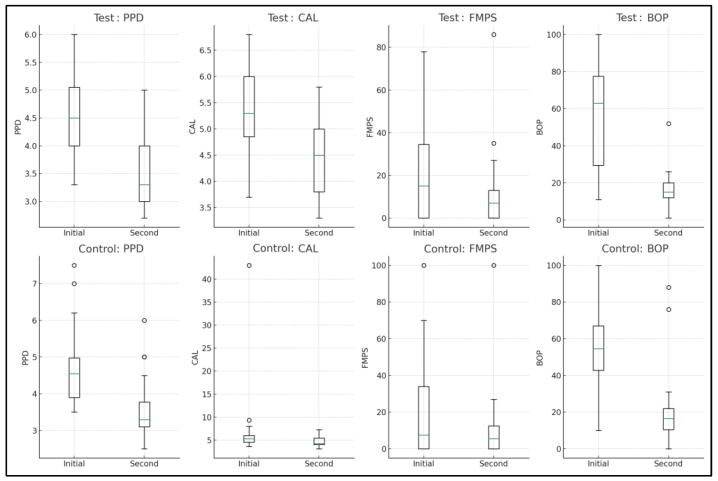
Intragroup comparison between baseline and 3-month measurements.

In the test group (n = 22), there were significant changes in PPD and BOP. The PPD median value reduced from 4.5 to 3.3 mm (*p* = 0.0005). Similarly, BOP showed a marked decrease, with the median dropping from 54.5% to 16.5% (*p* < 0.0001). However, changes in CAL and FMPS were not statistically significant for the test group, with the CAL median moving from −5.3 to −4.3 mm (*p* = 0.1583) and FMPS median changing from 7.5% to 5.5% (*p* = 0.2189) (Table 3, Figure 2).

**Figure 3:**
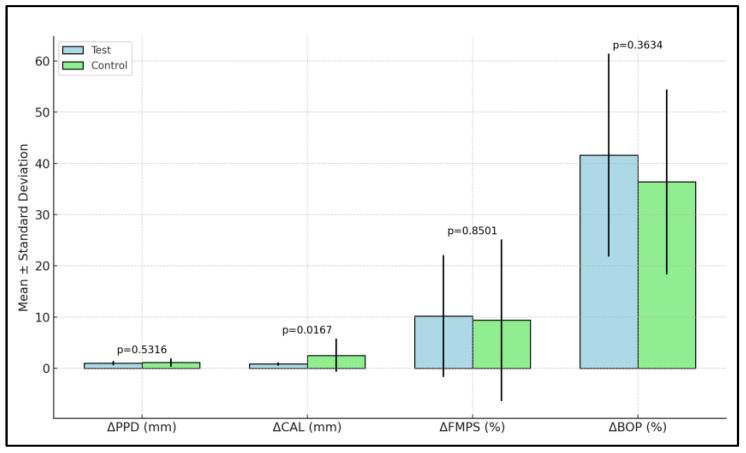
Modifications of clinical parameters between baseline and 3 months in the two study groups.

The change in PPD (ΔPPD) between baseline and three months was not statistically significant in either group. However, the change in CAL (ΔCAL) was statistically significant. The control group had a mean change of 0.81 mm, while the test group showed a greater change of 2.51 mm (*p* = 0.0167). ΔFMPS showed a reduction in both groups, but the difference was not statistically significant. Similarly, the change in BOP (ΔBOP), indicating a reduction in gingival inflammation, was not statistically significant between the groups (Table 4, Figure 3).

For the bacteria *Aa* and *Pi*, the changes in detection scores were not statistically significant. In the controls, 17% of patients showed a higher detection of *Aa*, compared to 4.5% in the test group, with similar percentages for lower detection and no change between the two groups. However, notable changes were observed in the detection of *Pg*, *Tf*, and *Td*. For *Pg*, a significant difference was found (*p* = 0.0346) with a higher percentage of lower detection in the test group (77%) compared to controls (39%). Only 4.5% of the test group showed higher detection of *Pg*, compared to 13% of controls, indicating a more substantial reduction in *Pg* in the test group. Similarly, significant findings were recorded for *Tf* (*p*-value = 0.0190) and *Td* (*p* = 0.0402). In the test group, a significant majority (86% for *Tf* and 63% for *Td*) showed lower detection in contrast to controls, where 47% and 26%, respectively, showed lower detection. However, the percentage of patients with no change in detection was also higher in the test group for both *Tf* and *Td* (Table 5).

The difference in variation in detection scores between the groups was not statistically significant for Aa and Pi. However, significant changes were observed for *Pg*, *Td*, and *Tf*, as shown in Table 6. 

Only the test group, consisting of 22 patients with stage III-IV periodontitis using a 0.2% sodium hypochlorite solution, was assessed via the VAS questionnaire. The overall statistical analysis of data resulting from the VAS questionnaires (n = 22) and the gender-based and age-based comparisons are included in Table 7, Table 8 and Table 9, respectively.

## 4. Discussion

To the best of our knowledge, this is the first study to clinically and microbiologically investigate the effects of a complex protocol involving low-cost potent antimicrobials for irrigation and home-care rinsing, combined with systemic antibiotics and antivirals used as an adjunct to subgingival instrumentation in step 2 of periodontal therapy [39]. The working hypotheses aimed to evaluate the outcome of this protocol, which prescribes highly effective disinfectant agents with minimal costs, both by the patient (as home care) and by the clinician (in the course of the periodontal treatment), to efficiently target all the microorganisms involved in the etiology of the disease (viral and bacterial).

Over the last few decades, the authors of the used protocol have adjusted the NaOCl concentrations (0.05%, 0.10%, 0.2%, 0.25%), considering the individual tolerance and acceptability of the taste, smell, and strength of the solutions by the patients [35,36,37,38]. As the highest tolerated concentration of the NaOCl solution found in the literature was 0.5% [40], we used 0.2% as an intermediate value within the range of concentrations suggested by the latest version of the protocol [17].

The complexity of the protocol made comparing the results of our study with similar studies difficult. However, in the past two decades, several concentrations of NaOCl have been proposed as antimicrobial adjuncts in the treatment of periodontal disease (5.6% to facilitate gingival curettage [28,30], 0.5% in subgingival irrigation [26,27], 0.05% [42], 0.1% (20) in an oral rinsing solution, or both subgingival irrigations and rinses with 0.25% solutions [24]). Periodontitis can be a risk factor for many systemic conditions due to the presence of bacteria in the bloodstream and pro-inflammatory proteins that can affect other parts of the body [21,47,48,49]. Regarding this, the effect of concomitant oral rising and subgingival irrigation with 10% PVP–iodine during subgingival instrumentation on the prevalence and intensity of bacteria activity was investigated in a recent study [50], which found significantly fewer bacteria of oral origin in blood samples that were taken from the central blood flow 3 min after periodontal instrumentation complemented with PVP irrigation when compared to the same treatment using tap water.

The therapeutic approach in the second step of periodontal therapy, as recommended by the recent EFP S3 level Clinical Practice Guideline for the Treatment of Stage I–III Periodontitis, blends mechanical therapy, which consists of debridement of the periodontal pockets by subgingival instrumentation with local anti-infective therapy in order to prevent plaque accumulation and to disinfect the root surfaces [15]. The protocol recommends subgingival instrumentation in two phases: on day 0, full-mouth gross scaling with an ultrasonic instrument, completed after ten days with scaling and root planing only in sites with verified calculus or suspicion of calculus. According to the authors, antiseptics and antibiotics will remove periodontal pathogens in sites that have not been scaled [39]. The term “root surface disinfection” was first referred to when topical antimicrobial agents such as chlorhexidine, peroxides, povidone–iodine (PVP–I), or sodium hypochlorite (NaOCl) were applied professionally as adjuncts to mechanical instrumentation [39]. Nevertheless, the use of local antiseptics is not without limitations, considering the fact that, to obtain bactericidal activity against many of the known periodontal pathogens, very high concentrations of many substances are required. At the same time, these irrigations cannot be used in patients with thyroid gland dysfunction and are contraindicated in individuals with thyroid dysfunction due to their potential impact on thyroid hormone syntheses, which limits the general use of this antiseptic [51,52].

The adjunctive use of antivirals in this protocol is justified by the fact that a bacteriologic cause alone seems insufficient in accounting for several clinical features of periodontal disease [7]. Many authors have stated that periodontal tissue destruction can be influenced by periodontal herpesviruses, human cytomegalovirus (HCMV), Epstein–Barr virus (EBV), and herpes simplex 1 virus in particular [7,35,53,54]. This mechanism is supposedly generated by a virally induced deterioration of the periodontal defense, which leads to the enhanced virulence of resident pathogenic bacteria by the virally mediated release of cytokines and chemokines from the host cells [36,55,56,57].

The increasing costs of periodontal healthcare combined with a growing number of low-income populations worldwide highlight the need to assess different approaches in the management of periodontal disease [21,49,58]. Especially in the numerous low-income populations around the globe, patients must be capable of managing their chronic disease using an efficient and cost-effective antiseptic agent. Periodontal disease (both gingivitis and periodontitis) is in fact the most common global disease, alongside dental caries [21,49,59]. The prevalence of periodontitis has remained largely unchanged over the last 25 years and, in addition to the dentition-related condition (tooth loss), there is a high association with several comorbidities that have a major impact on overall systemic health [21,49]. Periodontitis is widely preventable, but its therapeutic approach lacks the funding for prevention and treatment, especially in low- and middle-income countries where the cost of the therapy often exceeds available resources [49].

Affordable and highly effective therapy is needed to provide successful results and life-long maintenance for periodontitis patients. These goals are achieved when combining classic subgingival instrumentation with the powerful antiseptics used in eradicating various medical infections, with PVP–I and NaOCl being the best examples [60,61,62,63,64] along with oral treatments [36,39,50,65,66,67,68,69,70,71].

As recommended by the clinical practice guidelines, the adjunctive use of specific systemic antibiotics may be considered for specific patient categories, like stage III periodontitis, while considering the global concerns regarding the overuse of antibiotics and the development of antibiotic resistance [15]. A recent meta-analysis revealed a statistically significantly improved outcome after the adjunctive use of systemic antibiotics in the second step of periodontal therapy, with the metronidazole and amoxicillin association being the most effective on the clinical outcomes [72]. Our clinical findings show that after 3 months, significant improvements were observed in the control group across all measured variables. However, even the control group experienced a mean change of 0.81 mm in CAL gain, while the test group showed a more substantial change of 2.51 mm (*p* = 0.016), suggesting that this group experienced a greater improvement in terms of attachment-level gain compared to the controls, which is a strong indicator for periodontal treatment success. Therefore, the null hypothesis was rejected. To compare the effect of subgingival antimicrobial irrigations, in a randomized split-mouth study that clinically and microbiologically evaluated the periodontal results after subgingival irrigations with 10% PvP-I, no statistical difference between the control (subgingival irrigations with saline) and test groups was recorded, except for deep pockets at the 6-month re-evaluation and a slight continuous decrease in the number of viable anaerobic bacteria, with no statistical significance [73]. Another similar study investigating PvP-I subgingival irrigations in deep pockets obtained a 95% or greater reduction in total pathogen counts in 44% of pockets having more than 6 mm depth, and the reduction in mean pocket depth was greater than irrigations with saline [74].

The efficiency of the systemic administration of antibiotics was better emphasized in a recent clinical and microbiological study, which investigated 3- and 7-day antibiotic regimes that resulted in statistically significant improvements at 3 and 6 months after treatment. However, there were no statistically significant differences between the treatment groups, suggesting that in patients with stage III/IV, grade C periodontitis, adjunctive systemic antibiotics taken for only 3 days can yield improvements similar to a 7-day regime [75].

When comparing the therapy steps to the clinical practice guidelines, the distinctiveness of the present study is found in the patient’s intake of antiviral medication during the first step of therapy. The sequence of the therapeutical steps, as recommended by the recent clinical practice guideline, generally reflects the proposed protocol (interventions aimed at removing supragingival plaque and calculus, subgingival instrumentation, the use of adjunctive physical or chemical agents, and the use of adjunctive systemic antimicrobials) [15]. However, several differences can be noted: full-mouth gross scaling at day 0 to reduce subgingival calculus and thus facilitate the detection of calculus in the next appointment after ten days and address the viral implication—another component of the etiology of periodontal disease which is usually neglected. Herpesviruses trigger bacterial upgrowth; therefore, the adjunctive administration of antivirals is efficient against viruses in the lytic phase, so it makes sense that the antiviral therapy is carried out first [36,55,76]. Furthermore, on day 10, this protocol recommends SRP only in sites with verified calculus or suspicion of calculus, on the assumption that antiseptics and antibiotics will remove periodontal pathogens in sites that have not been scaled. In this protocol, antibiotics (amoxicillin + metronidazole or ciprofloxacin + metronidazole) are prescribed in all cases of severe periodontitis, with no prior periodontal pathogen identification.

The overall patient-centered data in our study revealed that unpleasant taste perception had a median value of 6, indicating moderate taste discomfort. The intolerance level was higher, with a median of 7, showing low acceptability. Tooth staining and dry mouth sensation, with medians of 4 and 5, respectively, suggest mild to moderate side effects. The ease of solution preparation and economic impact were favorable, with medians of 5 and 1, highlighting user-friendliness and low cost. Minimal mucosal irritation and long-term negative effects, both with low median scores of 1, along with cleaner-feeling teeth (median of 5), indicate the solution’s effectiveness and tolerability, despite some sensory drawbacks.

The findings in the gender-based analysis of perception differences in the use of the sodium hypochlorite rinsing solution revealed no statistically significant differences between men and women across various investigated aspects. This uniformity suggests the solution’s effects and acceptability are comparable across genders, reinforcing its broad applicability for dental hygiene practices without gender-specific biases or outcomes.

The analysis of the age-based comparison of various factors associated with the use of the sodium hypochlorite home-care rinsing divided participants into two groups: younger (n = 8) and older (n = 14). Notable findings include a significant difference in unpleasant taste perception, with younger participants (below 45 years) reporting a higher mean score (7.00 ± 1.07) compared to the older group (5.92 ± 1.12, *p* = 0.038). Similarly, tolerance during use was rated significantly higher by younger participants (7.25 ± 0.71) than by their older counterparts (6.08 ± 1.26, *p* = 0.026). In contrast, variables such as tooth staining intensity after 3 months, dry mouth sensation intensity, ease of solution preparation, and mucosal irritation showed no significant age-related differences. A significant variation was observed in the changes in taste after use, where younger participants experienced a higher mean change (3.50 ± 1.20) compared to the older group (2.33 ± 0.98, *p* = 0.021). However, the economic impact was uniformly reported as low across both groups, with no variation. The variables of long-term negative effects and cleaner-feeling teeth after rinsing did not show significant differences between the age groups. This comparison underscores specific age-related perceptions and tolerances of the 0.2% hypochlorite rinsing solution, highlighting the importance of considering age as a factor in evaluating product efficacy and user satisfaction.

The effectiveness of the used protocol is underlay in the broad approach of periodontal disease, which is infectious (both viral and bacterial) and progressive. It represents a highly effective response to the poorly managed stages of periodontitis and to patients who do not respond favorably to other protocols.

The limitations of the present study are represented by the relatively small differences between the two groups at the 3-month recall. This can be attributed to the randomization and to the initial values of the clinical parameters in the test group, with mean values corresponding to more severe stages of periodontitis. Another limitation is the lack of studies in the literature using this protocol. The adequacy of the PCR technique for microbial frequency detection can also be questioned, as nowadays, there are newer genomic methods to identify periodontal bacteria. At first glance, the protocol might appear difficult to implement, both for the patient and the clinician, due to the complex timing of administration and the variety of substances used; this perception fades with the analysis of the patient-centered results.

Future research directions should also include possible modifications of the taste and smell of the home-care antiseptic oral rinse to increase its tolerance and acceptability in all categories of periodontal patients. Nevertheless, the main future research direction generated by our study is the investigation of the separate role of each antimicrobial substance in achieving positive treatment results.

## 5. Conclusions

Despite its complexity, the proposed cause-related protocol for the treatment of severe and advanced periodontal disease, including a strong multifactorial, anti-infective approach (mechanic, antiseptic, antibiotic, and antiviral), resulted in substantial improvements in the clinical and microbiological parameters when compared with the current antimicrobial recommendations. Additionally, the proposed protocol has the advantage of affordability for low-income populations in which severe/advanced forms of periodontal disease represent a problem that is difficult to manage. Further clinical studies are needed to elucidate the stability and the long-term results of this approach and the separate role of each antimicrobial in achieving these results.

## Figures and Tables

**Table 1:** Patient demographics.

Variables	Control (n = 23)	Test (n = 22)	*p*-Value *
Age, years (mean ± SD)	48.3 ± 9.1	43.2 ± 8.7	0.061 ^t^
Age range	34–71	33–63	-
Sex (n%)			0.180
Men	14 (61%)	9 (41%)	
Women	9 (39%)	13 (59%)	

*—calculated using the chi-square test, unless otherwise specified; ^t^—calculated using Student’s *t*-test; SD—standard deviation.

**Table 2:** Baseline and 3-month measurements (intergroup comparison).

Variables	Baseline Measurements (Intergroup Comparison)			3-Month Measurements (Intergroup Comparison)		
	Control (n = 23)	Test (n = 22)	*p*-Value *	Control (n = 23)	Test (n = 22)	*p*-Value *
PPD (mm), mean ± SD	4.52 ± 0.71	4.70 ± 1.09	0.513 ^t^	3.53 ± 0.79	3.60 ± 0.86	0.777 ^t^
CAL (mm), mean ± SD	−5.31 ± 0.81	−7.12 ± 8.12	0.293 ^t^	−4.50 ± 0.81	−4.61 ± 1.13	0.708 ^t^
FMPS (%)	22.13 ± 24.96	21.11 ± 28.21	0.898 ^t^	11.96 ± 18.95	11.73 ± 21.63	0.969 ^t^
BOP (%)	58.17 ± 29.94	57.64 ± 23.08	0.947 ^t^	16.57 ± 9.75	21.23 ± 21.10	0.343 ^t^
*Aa*			0.387			0.224
Undetectable	14 (61%)	16 (73%)		16 (70%)	19 (86%)	
Mild Detection	1 (4%)	0 (0%)		1 (4%)	2 (9%)	
Moderate Detection	4 (17%)	1 (5%)		4 (17%)	1 (5%)	
High Detection	4 (17%)	5 (23%)		1 (4%)	0 (0%)	
*Pg*			0.384			0.021
Undetectable	7 (30%)	5 (23%)		10 (43%)	19 (86%)	
Mild Detection	1 (4%)	2 (9%)		5 (22%)	2 (9%)	
Moderate Detection	12 (52%)	8 (36%)		5 (22%)	1 (5%)	
High Detection	3 (13%)	7 (32%)		3 (13%)	0 (0%)	
*Pi*			0.432			0.166
Undetectable	14 (61%)	12 (55%)		16 (70%)	15 (68%)	
Mild Detection	4 (17%)	5 (23%)		4 (17%)	5 (23%)	
Moderate Detection	5 (22%)	3 (14%)		3 (13%)	2 (9%)	
High Detection	0 (0%)	2 (9%)		0 (0%)	0 (0%)	
*Tf*			0.865			<0.0001
Undetectable	2 (9%)	2 (9%)		4 (17%)	14 (64%)	
Mild Detection	1 (4%)	2 (9%)		5 (22%)	3 (14%)	
Moderate Detection	4 (17%)	5 (23%)		3 (13%)	5 (23%)	
High Detection	16 (70%)	13 (59%)		9 (39%)	0 (0%)	
*Td*			0.531	3.53 ± 0.79	3.60 ± 0.86	<0.0001
Undetectable	5 (22%)	5 (23%)		−4.50 ± 0.81	−4.61 ± 1.13	
Mild Detection	10 (43%)	6 (27%)		11.96 ± 18.95	11.73 ± 21.63	
Moderate Detection	8 (35%)	10 (45%)		16.57 ± 9.75	21.23 ± 21.10	
High Detection	0 (0%)	1 (5%)				

*—calculated using the Chi-square test, unless otherwise specified; ^t^—calculated using Student’s *t*-test; SD—standard deviation; PPD—periodontal pocket depth; CAL—clinical attachment level; FMPS—full-mouth plaque score; BOP—bleeding on probing; *Aa*—*Aggregatibacter actinomycetemcomitans*; *Pi*—*Prevotella intermedia*; *Pg*—*Porphyromonas gingivalis*; *Tf*—*Tannerella forsythia*; *Td*—*Treponema denticola*.

**Table 3:** Intragroup comparison between baseline and 3 months.

Variables	Baseline	3 Months	*p*-Value *
Group 1 (n = 23)			
PPD (mm), median (IQR)	4.5 (4.0–5.05)	3.3 (3.0–4.0)	<0.0001
CAL (mm), median (IQR)	−5.3 (4.85–6.0)	−4.5 (3.8–5.0)	<0.0001
FMPS (%), median (IQR)	15.0 (0.1–34.5)	7.0 (0.1–13.0)	0.036
BOP (%), median (IQR)	63.0 (29.5–77.5)	15.0 (12.0–20.0)	<0.0001
Group 2 (n = 22)			
PPD (mm), median (IQR)	4.5 (3.9–4.97)	3.3 (3.1–3.77)	0.0005
CAL (mm), median (IQR)	−5.3 (4.55–6.0)	−4.3 (4.0–5.45)	0.158
FMPS (%), median (IQR)	7.5 (0.1–34.0)	5.5 (0.1–12.5)	0.218
BOP (%), median (IQR)	54.5 (42.7–67.0)	16.5 (10.5–22.0)	<0.0001

*—calculated using the Mann–Whitney U test; IQR—interquartile range; PPD—periodontal pocket depth; CAL—clinical attachment level; FMPS—full-mouth plaque score; BOP—bleeding on probing.

**Table 4:** Modifications of clinical parameters between baseline and 3 months.

Variables (Mean ± SD)	Control (n = 23)	Test (n = 22)	*p*-Value *
ΔPPD (mm)	0.99 ± 0.43	1.10 ± 0.80	0.531
ΔCAL (mm)	0.81 ± 0.31	2.51 ± 3.26	0.016
ΔFMPS (%)	10.17 ± 11.90	9.38 ± 15.79	0.850
ΔBOP (%)	41.61 ± 19.81	36.41 ± 18.07	0.363

*—calculated using Student’s *t*-test; SD—standard deviation; PPD—periodontal pocket depth; CAL—clinical attachment level; FMPS—full-mouth plaque score; BOP—bleeding on probing.

**Table 5:** Modifications of detection scores of investigated pathogens in the test and control groups.

Variables	Control (n = 23)	Test (n = 22)	*p*-Value *
*Aa*			0.382
Higher detection	4 (17.39%)	1 (4.55%)	
Lower detection	6 (26.09%)	6 (27.27%)	
No change	13 (56.52%)	15 (68.18%)	
*Pg*			0.034
Higher detection	3 (13.04%)	1 (4.55%)	
Lower detection	9 (39.13%)	17 (77.27%)	
No change	11 (47.83%)	4 (18.18%)	
*Pi*			0.764
Higher detection	5 (21.74%)	3 (13.64%)	
Lower detection	8 (34.78%)	9 (40.91%)	
No change	10 (43.48%)	10 (45.45%)	
*Tf*			0.019
Higher detection	5 (21.74%)	2 (9.09%)	
Lower detection	11 (47.83%)	19 (86.36%)	
No change	7 (30.43%)	1 (4.55%)	
*Td*			0.040
Higher detection	4 (17.39%)	2 (9.09%)	
Lower detection	6 (26.09%)	14 (63.64%)	
No change	13 (56.52%)	6 (27.27%)	

*—calculated using the chi-square test; *Aa*—*Aggregatibacter actinomycetemcomitans*; *Pi*—*Prevotella intermedia*; *Pg*—*Porphyromonas gingivalis*; *Tf*—*Tannerella forsythia*; *Td*—*Treponema denticola*.

**Table 6:** Variation in detection frequency scores of pathogens between baseline and 3 months.

Variables	Control Group Variation (Min–Max (Median))	Test Group Variation (Min–Max (Median))	*p*-Value *
*Aa*	−4.0–4.0 (0.0)	−4.0–2.0 (0.0)	0.368
*Pg*	−3.0–3.0 (0.0)	−3.0–2.0 (−2.0)	0.004
*Pi*	−2.0–2.0 (0.0)	−2.0–2.0 (0.0)	0.532
*Td*	−2.0–1.0 (0.0)	−3.0–1.0 (−1.0)	0.009
*Tf*	−3.0–2.0 (0.0)	−4.0–2.0 (−2.0)	0.016

*—calculated using the Mann–Whitney U test; *Aa*—*Aggregatibacter actinomycetemcomitans*; *Pi*—*Prevotella intermedia*; *Pg*—*Porphyromonas gingivalis*; *Tf*—*Tannerella forsythia*; *Td*—*Treponema denticola*.

**Table 7:** Overall statistical analysis of data resulting from the VAS questionnaires (n = 22).

Variables	Median	IQR	Mean	SD
Unpleasant taste perception	6	2	6.33	1.2
Tolerance during use	7	1	6.52	1.21
Tooth staining intensity after 3 months	4	1	3.62	0.8
Dry mouth sensation intensity	5	1	4.5	0.89
Ease of solution preparation	5	0	4.9	0.31
Changes in taste after use	3	1.25	2.8	1.2
Economic impact	1	0	1	0
Mucosal irritation	1	1	1.55	1
Long-term negative effects	1	0	1.35	0.93
Teeth felt cleaner after rinsing	5	1	4.55	0.6

IQR—interquartile range; SD—standard deviation.

**Table 8:** Gender-based comparison.

Variables (Mean ± SD)	Men (n = 12)	Women (n = 10)	*p*-Value *
Unpleasant taste perception	6.25 ± 1.29	6.44 ± 1.13	0.720
Tolerance during use	6.42 ± 1.38	6.67 ± 1.00	0.638
Tooth staining intensity after 3 months	3.83 ± 0.83	3.33 ± 0.71	0.149
Dry mouth sensation intensity	4.45 ± 0.93	4.56 ± 0.88	0.780
Ease of solution preparation	4.91 ± 0.30	4.89 ± 0.33	0.883
Changes in taste after use	3.00 ± 1.18	2.56 ± 1.24	0.402
Economic impact	1.00 ± 0.00	1.00 ± 0.00	-
Mucosal irritation	1.64 ± 1.21	1.44 ± 0.73	0.652
Long-term negative effects	1.45 ± 1.21	1.22 ± 0.44	0.575
Teeth felt cleaner after rinsing	4.45 ± 0.69	4.67 ± 0.50	0.411

*—calculated using Student’s *t*-test; SD—standard deviation.

**Table 9:** Age-based comparison.

Variables (Mean ± SD)	Younger (n = 8)	Older (n = 14)	*p*-Value *
Unpleasant taste perception	7.00 ± 1.07	5.92 ± 1.12	0.038
Tolerance during use	7.25 ± 0.71	6.08 ± 1.26	0.026
Tooth staining intensity after 3 months	3.50 ± 0.93	3.69 ± 0.75	0.605
Dry mouth sensation intensity	4.38 ± 0.92	4.58 ± 0.90	0.624
Ease of solution preparation	4.75 ± 0.46	5.00 ± 0.10	0.607
Changes in taste after use	3.50 ± 1.20	2.33 ± 0.98	0.021
Economic impact	1.00 ± 0.00	1.00 ± 0.00	-
Mucosal irritation	1.62 ± 1.41	1.50 ± 0.67	0.788
Long-term negative effects	1.52 ± 1.33	1.17 ± 0.39	0.362
Teeth felt cleaner after rinsing	4.25 ± 0.71	4.75 ± 0.45	0.055

*—calculated using Student’s *t*-test; SD—standard deviation.

## Data Availability

The data presented in this study are available on request from the corresponding author.
